# Effects of *Spartina alterniflora* Invasion on Soil Microbial Community Structure and Ecological Functions

**DOI:** 10.3390/microorganisms9010138

**Published:** 2021-01-09

**Authors:** Minmin Cao, Lina Cui, Huimin Sun, Xiaomian Zhang, Xiang Zheng, Jiang Jiang

**Affiliations:** 1 Collaborative Innovation Center of Sustainable Forestry in Southern China of Jiangsu Province, Nanjing Forestry University, Nanjing 210037, China; healer_cao@outlook.com (M.C.); Selina_cui@outlook.com (L.C.); Xiang-Zheng@outlook.com (X.Z.); 2 Ministry of Education Key Laboratory for Biodiversity Science and Ecological Engineering, National Observation and Research Station for Yangtze Estuarine Wetland Ecosystems, Institute of Biodiversity Science, School of Life Sciences, Fudan University, 2005 Songhu Road, Shanghai 200438, China; huimin_sun@outlook.com; 3 Zhejiang Forestry Academy, Hangzhou 310023, China; Amanda-zhangxm@outlook.com

**Keywords:** soil microbial, community structure, high throughput sequencing, *Spartina alterniflora* invasion, mangrove, ecological functions, tax4fun analysis, funguild analysis

## Abstract

It has been reported that the invasion of *Spartina alterniflora* changed the soil microbial community in the mangrove ecosystem in China, especially the bacterial community, although the response of soil fungal communities and soil microbial ecological functions to the invasion of *Spartina alterniflora* remains unclear. In this study, we selected three different communities (i.e., *Spartina alterniflora* community (SC), *Spartina alterniflora*–mangrove mixed community (TC), and mangrove community (MC)) in the Zhangjiangkou Mangrove Nature Reserve in China. High-throughput sequencing technology was used to analyze the impact of *Spartina alterniflora* invasion on mangrove soil microbial communities. Our results indicate that the invasion of *Spartina alterniflora* does not cause significant changes in microbial diversity, but it can alter the community structure of soil bacteria. The results of the LEfSe (LDA Effect Size) analysis show that the relative abundance of some bacterial taxa is not significantly different between the MC and SC communities, but different changes have occurred during the invasion process (i.e., TC community). Different from the results of the bacterial community, the invasion of *Spartina alterniflora* only cause a significant increase in few fungal taxa during the invasion process, and these taxa are at some lower levels (such as family, genus, and species) and classified into the phylum *Ascomycota*. Although the invasion of *Spartina alterniflora* changes the taxa with certain ecological functions, it may not change the potential ecological functions of soil microorganisms (i.e., the potential metabolic pathways of bacteria, nutritional patterns, and fungal associations). In general, the invasion of *Spartina alterniflora* changes the community structure of soil microorganisms, but it may not affect the potential ecological functions of soil microorganisms.

## 1. Introduction

Mangroves are one of the most important ecosystems in tropical and subtropical coastal wetlands, and they play an important role in maintaining high productivity and biodiversity and providing stable habitats for organisms [1,2,3]. Due to the impact of a large number of human activities in recent years (e.g., emission of nitrogen- and phosphorus-rich wastewater, pollution of organic and heavy metals, over-harvesting of mangrove resources, and invasion of exotic species, etc.), mangrove ecosystem services and their values are facing serious threats [4,5,6]. Plant invasion has gradually become an emerging driver of global change [7], and mangroves are one of the most threatened ecosystems in the world [8]. The invasion of exotic plants not only modifies coastal hydrology and geomorphology of the mangrove ecosystem, increases fire hazard by increasing fuel load, alters light regimes, soil fertility, nutrient fluxes and biogeochemical cycles, particularly ecosystem carbon (C) and nitrogen (N) cycles, but also causes significant changes in the mangrove plants and animals community structure, diversity, and abundance [9,10,11].

Chinese mangrove ecosystems are vulnerable to the invasive species *Spartina alterniflora* (a perennial herb), which is native to North America and was introduced to China in 1979 to accelerate the deposition and stabilization of tidal flats [12]. In the past few decades, *Spartina alterniflora* has become one of the dominant species in the coastal wetlands in China due to its unique physiological and ecological characteristics (e.g., higher growth rate, higher net primary yield, and higher salt tolerance) compared to native plants [13,14]. So far, *Spartina alterniflora* has spread throughout the coastline of mangrove wetlands [15], thus greatly affecting the mangrove ecosystem in China. *Spartina alterniflora* invasion has been demonstrated to significantly change the accumulation and turnover of soil organic carbon (C) and nitrogen (N) in mangrove wetlands [16] as well as their soil physicochemical properties [10,17]. Moreover, studies have also shown that *Spartina alterniflora* invasion changes greenhouse gas fluxes in mangrove wetlands by controlling soil conductivity, microbial biomass, microbial respiration, etc. [18]. It was reported that *Spartina alterniflora* invasion not only changed the dominant species and biodiversity of macrobenthos in mangrove wetlands [19] but also had a significant impact on soil bacterial abundance and community structure [20]. Although extensive research has been conducted to investigate the effects of *Spartina alterniflora* invasion on mangrove ecosystems [16,17,18], the elucidation of soil microbial communities responses to *Spartina alterniflora* invasion remains limited [20,21].

Soil microbes are one of the important factors of soil fertility, which can control the turnover and formation of soil organic matter (SOM) by decomposing various organic detritus. Therefore, soil microbes play a vital role in soil material transformation and energy flow [22,23]. Soil microbial communities are significantly affected by various environmental factors, such as plant communities, soil physicochemical properties, and soil nutrient substrate, etc. [21,24]. Plant communities can directly or indirectly influence soil microbial communities by altering the quantity and quality of litter [24,25,26]. Soil physicochemical properties (e.g., soil pH and soil water content) have been considered as one of the important driving factors of soil microbial communities [21,27]. For example, soil pH can regulate soil microbial communities by selecting species with compatible growth strategies, changing the growth and proliferation of soil microorganisms, and affecting the effectiveness of soil nutrients [28,29,30]. Moreover, it has been reported that the quality and quantity of soil nutrient substrates can alter soil microbial communities [31]. Previous studies have also found that soil microbial communities are closely related to soil organic matter (SOM), soil organic carbon (SOC), total nitrogen (TN), etc. [20,21,31]. It has been reported that one of the main reasons for the successful invasion of invasive plants is to promote the microbial community structure of the rhizosphere soil, improve the function of the microbial community, and thereby creating a better soil microenvironment, which in turn aggravates the invasion process [32,33,34]. Therefore, exploring the impact of *Spartina alterniflora* invasion on the soil microbial abundance and diversity, community structure, and functional groups in mangrove wetlands will help better understand the influential mechanisms of *Spartina alterniflora* invasion on mangrove ecosystems.

It was reported that *Spartina alterniflora* invasion modified the soil microbial communities [35], particularly the bacterial communities [21,36], including several specific soil microbial taxa associated with nitrification [37]. However, the research on *Spartina alterniflora* invasion on soil microbial in mangrove ecosystems is still limited, and the current research is mainly focused on the diversity and community structure of bacterial communities [20,21]. In contrast, the response of soil fungal communities and soil microbial ecological functions to the invasion of *Spartina alterniflora* remains unclear. In this study, we selected three different communities (i.e., *Spartina alterniflora* community (SC), *Spartina alterniflora*–mangrove mixed community (TC), and mangrove community (MC)) in the Zhangjiangkou subtropical Mangrove Nature Reserve in China using the research approach of space-for-time [38]. High-throughput sequencing technology was used for analysis to address the following problems: (i) How does the invasion of *Spartina alterniflora* affect the diversity and structure of soil bacterial and fungal communities? (ii) What functional groups in the soil bacterial and fungal communities have been affected by the invasion of *Spartina alterniflora*? Assessing the impact of *Spartina alterniflora* invasion on soil bacterial and fungal communities in mangrove ecosystems can provide a microbiological basis for studying the impact of *Spartina alterniflora* invasion on mangrove ecosystems.

## 2. Materials and Methods

### 2.1. Sampling Sites Description

This study was conducted in the subtropical Zhangjiang Estuary Mangrove National Nature Reserve (23°53′–23°57′ N, 117°23′–117°30′ E), Zhangzhou, Fujian Province in southeastern China. The reserve is located at the mouth of the Zhangjiang River and has a subtropical marine monsoon climate. The mean annual temperature of this reserve is 21.2 °C and the annual mean rainfall was 1714.5 mm, most of which occurs from April to September. The soil of this reserve contains a high salt content (generally above 1%). There are 13 clusters including the *Aegiceras corniculatum* forest, and the dominant mangrove plants are *Kandelia obovata*, *Avicennia marina*, and *Bruguiera gymnorrhiza*. The canopy height is 3–5 m. The protected area is rich in wetland resources with typical and representative ecological environments.

### 2.2. Experimental Design and Soil Sampling

For the sampling, we selected three transect lines that spanned the mangrove—salt marsh ecotone within the intertidal zone. The Uninvaded mangrove community (MC), the *Spartina alterniflora*–mangrove mixed community (TC), and the *Spartina alterniflora* community (SC) fully occupied by *Spartina alterniflora* were selected from sea to land in Zhangjiangkou National Mangrove Wetland Nature Reserve in July 2017. The specific direction and location information of transects has been described in detail in our previous study [10]. For each of the three transect lines, the above three types of sample plots were selected, and three sample points were selected for each sample plot (distributed in a zigzag pattern, and the distance between each two sampling points was 5–10 m). Two layers of samples (0–15, 15–30 cm) were taken in layers with an earth drill, and a total of 18 samples were obtained. All the samples were put in the ice box and transported to the laboratory for subsequent analysis.

### 2.3. Soil Properties Analysis

The −70 ℃ ultra-low temperature refrigerator was used to store soil samples for microbiological analysis. We dried other soil samples to a constant weight in an oven below 60 ℃ to measure the soil moisture content. The roots were picked out from the soil, and the soil was filtered with a 2-mm sieve to remove plant residues and stones [39]. The pH values of soil samples were determined by pH meter (PHS-3D, Rex, Shanghai, China). We used dilute HCl (5%) to acidify the air-dried sub-sample to achieve the purpose of removing carbonate. The procedure to extract fractionation of particulate organic carbon (POC) was referenced from Sun et al. [10]. Then, the POC and SOC content of the extracted soil and air-dried soil were measured by an element analyzer (Vario MACRO cube, Elementar, Langenselbold, Germany). The MOC content was obtained by subtracting the particulate organic carbon (POC) content from the total soil organic carbon content (i.e., SOC).

### 2.4. DNA Extraction and Polymerase Chain Reaction Amplifification

The CTAB/SDS method was used to extract total DNA from each soil sample by a PowerSoil DNA extraction kit (Mo Bio Laboratories Inc., Carlsbad, CA, USA), and the same sample was extracted three times in total. The NanoPhotometer spectrophotometer was used to detect the purity of the sample, and the Qubit v. 2.0 Flurometer was used to detect the concentration of the DNA sample, and 1% agarose gels was used to detect whether the DNA sample was degraded and contained impurities. A fragment covering the V3 + V4 region in the 16S rDNA gene and a fragment covering the ITS1 region in the ITS rDNA gene were selected to construct bacterial and fungal community libraries. In the 16S rDNA gene region, the primer 341F (5′-CCTACGGG NGGCWGCAG-3′) and 805R (5′-GACTACHVGGGT ATCTAATCC-3′) amplify the V3 + V4 region, and the BITS (5′-ACC TGCGGARGGATCA-3′) and B58S3 (5′-GAGATCCRTTGYTRAAAGTT-3′) primers amplify in the ITS1 region [10]. All polymerase chain reaction (PCR) reactions were carried out in 30 μL reactions with 15 μL of Phusion High-Fidelity PCR Master Mix (New England Biolabs), 0.2 μM of forward and reverse primers, and about 10 ng template DNA. Reaction conditions consisted of an initial 95 °C for 2 min, followed by 35 cycles of 95 °C for 30 s, 55 °C for 30 s, 72 °C for 60 s, and a final extension of 72 °C for 5 min.

PCR products were mixed in equidensity ratios. Then, the mixture PCR products were purified with GeneJET Gel Extraction Kit (Thermo Scientific, San Diego, CA, USA). Sequencing libraries were generated using NEB Next Ultra DNA Library Prep Kit for Illumina (New England Biolabs, MA, USA) following manufacturer’s recommendations and index codes were added. The library quality was assessed on the Qubit@ v. 2.0 Fluorometer (Life Technologies, Carlsbad, CA, USA) and Agilent Bioanalyzer 2100 system. Finally, the library was sequenced on an Illumina HiSeq2500 using the 250 paired-end protocol.

For the sequence obtained by sequencing, we completed data filtering by removing low-quality bases, Ns (the number of Reads with N ratio greater than 5%), and linker-contaminated sequences, and obtained credible target sequences for subsequent analysis. For the filtered sequence, we spliced the corresponding sequence fragments of paired-end sequencing using the sequence splicing method PEAR [40] firstly, and then we removed the quality filtering of the original label according to the QIIME v. 1.8.0 quality control process for the spliced sequence [41,42,43]. After chimera removal, sequences were assigned to operational taxonomic units (OTUs) with 97% pairwise identity as the threshold. The RDP Classifier (V2.2) was used to select the most abundant sequence from each OTU as a representative sequence, and then the uclust method was used to compare the representative sequence to the sliva rRNA database (release_132) and the UNITE database (V8.2) to classify the bacterial and fungal OTU into species [44]. The above high-throughput sequencing work was carried out by a professional biotechnology company, Annoroad Gene Technology Co., Ltd. Beijing, China. The barcode information corresponding to samples’ information was reported in Supplementary Table S10. Raw sequences were deposited at the Sequence Read Archive (SRA) of the National Center for Biotechnology Information (NCBI) under project accession number PRJNA689656.

### 2.5. Information on Illumina HiSeq Data

A total of 503,185 high-quality bacterial sequences and 521,549 high-quality fungi sequences were generated across all samples after sequence de-noising and quality filtering. The average number of sequences per sample for bacteria was 27,955 ± 1375 (mean ± standard deviation) and ranged from 24,825 to 31,227 per sample. The average number of sequences per sample for fungi was 28,975 ± 466 (mean ± standard deviation) and ranged from 28,072 to 29,571 per sample.

### 2.6. Statistical Analysis

Any significant difference in soil physicochemical properties and microbiological data among different communities was determined by one-way ANOVA followed by Tukey’s test, considering difference statistically at *p* < 0.05. All data were subjected to the Shapiro–Wilk and Levene’s test before the difference significance analysis. Venn diagrams were constructed to show shared and unique OTUs with InteractiVenn (http://www.interactivenn.net, accessed on 7 March 2020) [45].Linear discriminant analysis (LDA) coupled with effect size (LEfSe; http://huttenhower.sph.harvard.edu/galaxy/root?tool_id=PICRUSt_normalize, accessed on 16 March 2020) was used to identify the bacterial/fungal taxonomic groups differentially represented between treatments [46]. The criterion for LEfSe was set as LDA > 3.5 with *p* < 0.05. Principal Co-ordinates Analysis (PCoA) was used to visualize the bacterial/fungal community structure and functional structure using relative abundances of OTUs or functional groups in statistical software Canoco v. 5.0 (Microcomputer Power, Ithaca NY, USA). In addition, the PerMANOVA test in the “Vagen” package in R software (v. 4.0.3) was used to test the significance of the difference between vegetative types in microbial community structure (*p* < 0.05) [47].

Tax4Fun analysis was used to assess the bacterial community potential function by R (v. 4.0.3) [48], which contains six functional groups, including Metabolism, Environmental Information Processing, Genetic Information Processing, Cellular Processes, Human Diseases, and Organismal Systems. In each group, the potential function was further assigned to a second level with more subgroups. FUNGuild analysis was used to assess the fungal community potential function (http://www.stbates.org/guilds/app.php, accessed on 9 July 2020), which divided fungi into three categories according to the nutritional pattern: Pathotroph, Symbiotroph, and Saprotroph. The analysis then further subdivided them into 12 sub-categories: Animal Pathogens, Arbuscular Mycorrhizal Fungi, Ectomycorrhizal Fungi, Ericoid Mycorrhizal Fungi, Foliar Endophytes, Lichenicolous Fungi, Lichenized Fungi, Mycoparasites, Plant Pathogens, Undefined Root Endophytes, Undefined Saprotrophs, and Wood Saprotrophs. It also includes three types of fungi with special morphology: Yeast, Facultative yeast, and Thallus [49]. Statistical analysis was conducted using SPSS 20.0.

## 3. Results

### 3.1. Soil Properties between SC, TC, and MC

The soil pH in the two soil layers showed a trend of SC > MC > TC, and the pH of the SC community was significantly greater than that of the TC community. The soil water content of the two layers showed a trend of TC > MC > SC, but only in the 15–30 cm soil layer was there significant difference. The electrical conductivity (EC), soil organic carbon (SOC), and particulate organic carbon (POC) in the two soil layers all showed the trend of MC > TC > SC, and the differences were significant at a *p* level of < 0.05 (Table S1).

### 3.2. Microbial Communities Diversity between SC, TC, and MC

For the bacterial community, the community richness and diversity exhibited no significant difference in the 0–15 cm soil layer (Table 1). Chao1, Sobs, and Shannon indices of SC and TC community in the 15–30 cm soil layer showed significant differences. The Chao 1, Sobs, and Shannon indices of SC community were higher than TC community at a *p* level of < 0.05.

For the fungal community, the Chao1, Sobs, and Shannon indices in 0–15 cm soil layer all showed the trend of MC > TC > SC, whereas the Simpson index in 0–15 cm soil layer and all the richness and diversity indices in 15–30 cm soil layer showed the trend of TC > MC > SC (Table 2). However, none of the differences were significant at a *p* level of < 0.05.

### 3.3. Bacterial Community Structure at the Taxonomic Level between SC, TC, and MC

**Figure 1:**
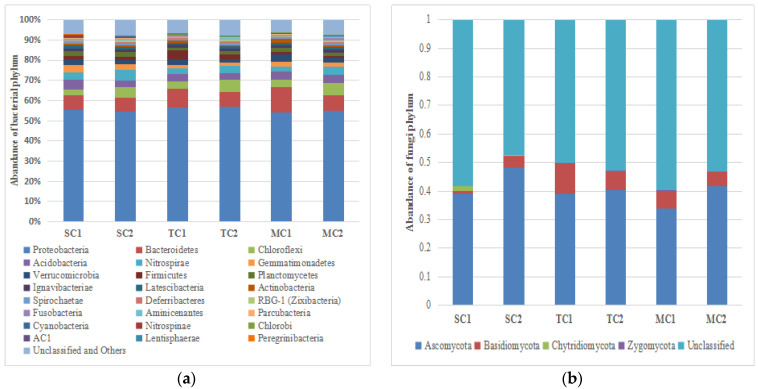
The relative abundance of bacteria (**a**) and fungi (**b**) phylum (% of the total number of reads) in the *Spartina alterniflora* community (SC), *Spartina alterniflora*–mangrove mixed community (TC), and the mangrove community (MC) samples, where 1 represents the 0–15 cm soil layer and 2 represents the 15–30 cm soil layer.

In the bacterial community, all sequences were classified to bacterial domain and assigned to 3948 OTUs across all samples, including 55 bacterial phyla, 122 classes, 143 orders, 215 families, and 301 genera. Proteobacteria (55.31% of the sequences and 36.42% of the OTUs) was the most abundant phylum, followed by Bacteroidetes (8.54% of the sequences and 9.15% of the OTUs), Chloroflexi (4.64% of the sequences and 7.24% of the OTUs), Acidobacteria (3.83% of the sequences and 5.25% of the OTUs), Nitrospirae (3.68% of the sequences and 1.83% of the OTUs), Gemmatimonadetes (2.40% of the sequences and 2.21% of the OTUs), Verrucomicrobia (2.39% of the sequences and 3.72% of the OTUs), Firmicutes (2.14% of the sequences and 2.79% of the OTUs), Planctomycetes (1.86% of the sequences and 7.06% of the OTUs), Ignavibacteriae (1.38% of the sequences and 1.16% of the OTUs), Latescibacteria (1.19% of the sequences and 2.29% of the OTUs), and Actinobacteria (1.06% of the sequences and 1.61% of the OTUs) (Figure 1a). A detailed list of phylum that includes the less abundant phyla (<1.0% of the sequences) is shown in Supplementary Table S2.

**Figure 2:**
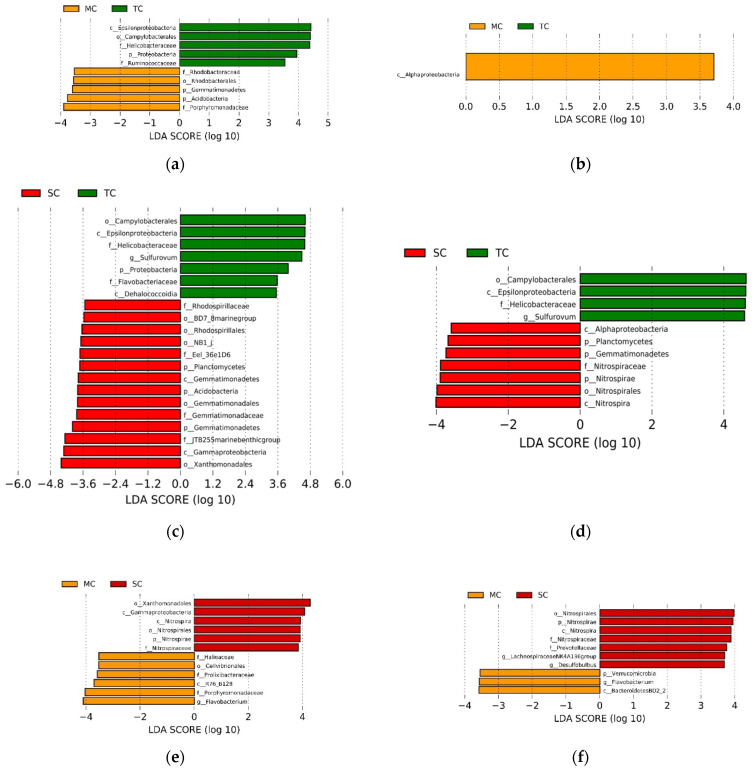
LEfSe analysis showing the significant differences at different bacterial taxonomic levels among SC, TC, and MC communities in 0–15 cm soil layer (**a**,**c**,**e**) and 15–30 cm soil layer (**b**,**d**,**f**). The colors of red, green, and yellow correspond to the SC, TC, and MC communities, respectively. Abbreviation: p—phylum; c—class; o—order; f—family; and g—genus.

The LEfSe analysis showed the abundance of some taxa differed among the SC, TC, and MC samples, respectively (LDA > 3.5, *p* < 0.05). When comparing the MC and TC communities, we found that the phylum Gemmatimonadetes and Acidobacteria, the order Rhodobacterales, and the family Rhodobacteraceae and Porphyromonadaceaei were more abundant in the MC community and the phylum Proteobacteria, the class Epsilonproteobacteria, the order Campylobacterales, and the family Helicobacteraceae and Ruminococcaceae had higher abundance in the TC community in the 0–15 cm soil layer; whereas only the class Alphaproteobacteria in MC community were significantly higher than TC community in the 15–30 cm soil layer (Figure 2a,b). When comparing the TC and SC communities, we observed that the phylum Proteobacteria, the class Epsilonproteobacteria and Dehalococcoidia, the order Campylobacterales, the family Helicobacteraceae and Flavobacteriaceae, and the genus *Sulfurovum* were more abundant in the TC community and the phylum Planctomycetes, Acidobacteria and Gemmatimonadetes, the class Gemmatimonadetes and Gammaproteobacteria, the order BD7_8 marine group, Rhodospirillales, NB1_j, Gemmatimonadales and Xanthomonadales, the family Rhodospirillaceae, Eel_36e1D6, Gemmatimonadaceae, and JTB255 marine benthic group had higher abundance in the SC community in the 0–15 cm soil layer; whereas the class Epsilonproteobacteria, the order Campylobacterales, the family Helicobacteraceae, and the genus *Sulfurovum* were more abundant in the TC community and the phylum Planctomycetes, Gemmatimonadetes and Nitrospirae, the class Alphaproteobacteria and Nitrospira, the order Nitrospirales, and the family Nitrospiraceae had higher abundance in the SC community in the 15–30 cm soil layer (Figure 2c,d). When comparing the MC and SC communities, we discovered that the class R76_B128, the order Cellvibrionales, the family Halieaceae, Prolixibacteraceae and Porphyromonadaceae, and the genus *Flavobacterium* were more abundant in the MC community and the phylum Nitrospirae, the class Gammaproteobacteria and Nitrospira, the order Xanthomonadales and Nitrospirales, and the family Nitrospiraceae had higher abundance in the SC community in the 0–15 cm soil layer; whereas the phylum Verrucomicrobia, the class BacteroidetesBD2_2, the genera *Flavobacterium* were more abundant in the MC community, the phylum Nitrospirae, the class Nitrospira, the order Nitrospirales, the family Nitrospiraceae and Prevotellaceae, and the genus *LachnospiraceaeNK4A136group* and *Desulfobulbus* had higher abundance in the SC community in the 15–30 cm soil layer (Figure 2e,f).

### 3.4. Fungal Community Structure at the Taxonomic Level between SC, TC and MC

In the fungal community, all sequences were classified to fungal domain and assigned to 2512 OTUs across all samples, including 4 fungal phyla, 18 classes, 50 orders, 85 families, 146 genera, and 188 species. Ascomycota (40.02% of the sequences and 24.44% of the OTUs) was the most abundant phylum, followed by Basidiomycota (5.59% of the sequences and 7.32% of the OTUs), Chytridiomycota (0.39% of the sequences and 0.36% of the OTUs), and Zygomycota (0.02% of the sequences and 0.20% of the OTUs) (Figure 1b). A detailed list of phyla is shown in Table S3.

**Figure 3:**
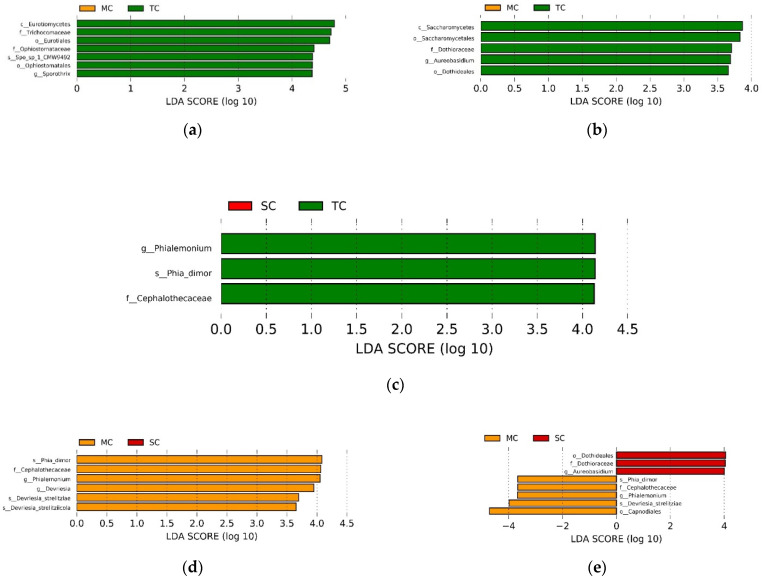
LEfSe analysis showing the significant differences at different fungal taxonomic levels among SC, TC, and MC communities in 0–15 cm soil layer (**a**,**b**,**d**) and 15–30 cm soil layer (**c**,**e**). The colors of red, green, and yellow correspond to the SC, TC, and MC communities, respectively. Abbreviation: p—phylum; c—class; o—order; f—family; g—genus; Phia_dimor—Phialemonium_dimorphosporum; and Spo_sp_1_CMW9492—Sporothrix_sp_1_CMW9492.

The LEfSe analysis showed the abundance of some taxa differed among the SC, TC, and MC samples, respectively (LDA > 3.5, *p* < 0.05). When comparing the MC and TC communities, we found that only the class Eurotiomycetes, order Eurotiales and Ophiostomatales, family Trichocomaceae and Ophiostomataceae, genus *Sporothrix* and species *Sporothrix_sp_1_CMW9492* in the TC community were significantly higher than in the MC community in the 0–15 cm soil layer; whereas only the class Saccharomycetes, order Saccharomycetales, and Dothideales, family Dothioraceae, and genus *Aureobasidium* in the MC community were significantly higher than in the TC community in the 15–30 cm soil layer (Figure 3a,b). When comparing the TC and SC communities, we observed that there was no significant difference between SC and TC communities at all taxa levels in the 0–15 cm soil layer, whereas only family Cephalothecaceae, genus *Phialemonium*, and species *Phialemonium_dimorphosporum* in the TC community were significantly higher than in the SC community (Figure 3c). When comparing the MC and SC communities, we discovered that only the family Cephalothecaceae, genus *Phialemonium* and *Devriesia*, and species *Phialemonium_dimorphosporum*, *Devriesia_strelitziae* and *Devriesia_strelitziicola* in the MC community was significantly higher than in the SC community in the 0–15 cm soil layer; whereas the order Dothideales, family Dothioraceae, and genus *Aureobasidium* were more abundant in the SC community in the 0–15 cm soil layer, the order Capnodiales, family Cephalothecaceae, genus *Phialemonium*, and species *Phialemonium_dimorphosporum* and *Devriesia_strelitziae* had higher abundance in the MC community in the 15–30 cm soil layer (Figure 3d,e).

### 3.5. Microbial Communities Structure at OTUs level

**Figure 4:**
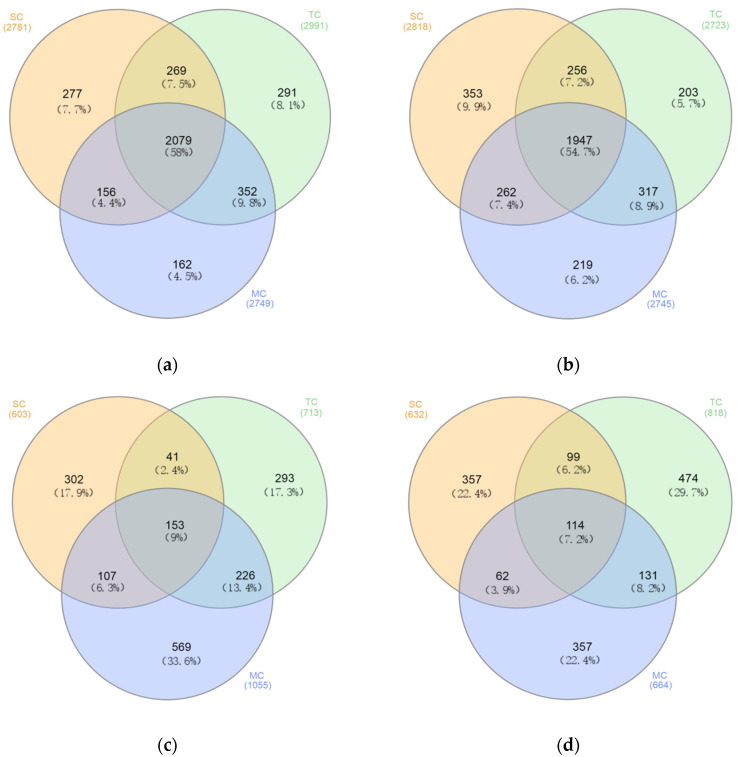
Venn diagram showing the unique and shared OTUs among the SC, TC, and MC samples in soil layer 0–15 cm (**a**,**c**) and 15–30 cm (**b**,**d**) in bacterial (**a**,**b**) and fungal (**c**,**d**) communities.

The number of shared and unique OTUs among the SC, TC, and MC samples in bacterial and fungal communities differed (Figure 4). The bacterial community shared half of the OTUs (58% in soil layer 0–15 cm and 54.7% in soil layer 15–30 cm) among the SC, TC, and MC samples (Figure 4a,b). Less than 10% of the OTUs (9% in soil layer 0–15 cm and 7.2% in soil layer 15–30 cm) were shared among the SC, TC, and MC samples in fungal community (Figure 4c,d).

**Figure 5:**
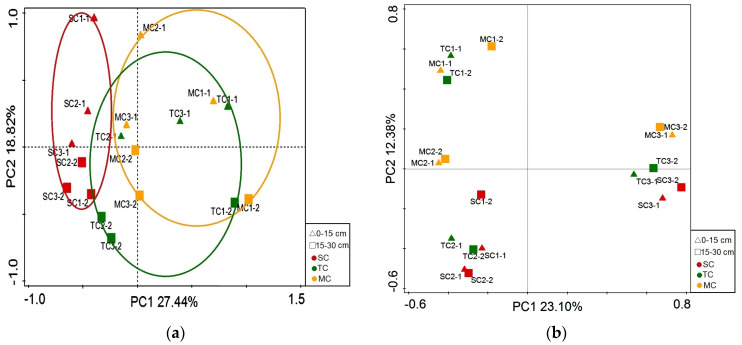
Results of PCoA showing the first two principal coordinates that combined explain 46.26% and 35.48% of the observed variation in bacterial (**a**) and fungal (**b**) community. The shapes of up triangle and square on the figure legend correspond to the soil layer of 0–15 cm and 15–30 cm, and the colors of red, green, and yellow correspond to the SC, TC, and MC communities, respectively. The ellipsoids corresponds to the differences among plant communities (PerMANOVA test, *p* < 0.05).

The first and second axes of PCoA analysis based on the OTUs data of bacterial community explained 27.44% and 18.82% of the variance, respectively (Figure 5a). However, using the OTUs data of fungal communities, the first and second axes of PCoA analysis explained 23.10% and 12.38% of the variance, respectively (Figure 5b).

### 3.6. Potential Metabolic Pathways of Soil Bacteria

A total of 3979 bacterial OTUs were included in the Tax4Fun analysis for functional assessment. A total of 6325 KEGG orthologues were found in the soil samples. In level 1, the group Metabolism had the highest abundance (60.21%), followed by Environmental Information Processing (18.38%), Genetic Information Processing (12.82%), Cellular Processes (5.75%), Human Diseases (1.68%), and Organismal Systems (1.08%). The top abundant functional pathways (relative abundance >1%) at level 2 was shown in Table S4. In 0–15 cm soil layer, only three Metabolic pathways in level 2, which are Replication and repair, Metabolism of cofactors and vitamins, and Endocrine system, show significant differences. However, there is no significant difference in the 27 Metabolism pathways in the 15–30 cm soil layer. PCoA analysis based on the functional assessment data variances among bacterial community with the first and second axes explaining 39.82% and 30.96% of the variance, respectively (Figure S1a).

### 3.7. Trophic Modes and Functional Groups of Soil Fungi

A total of 833 fungal OTUs were included in the FUNGuild analysis for functional assessment, but only 451 OTUs have been predicted for its ecological function. The OTUs were assigned to 7 trophic modes and 54 guilds, of which the trophic mode Saprotroph (10.33%) had the highest abundance, followed by Pathotroph-Saprotroph-Symbiotroph (4.96%), Pathotroph-Saprotroph (4.43%), Pathotroph (2.78%), Symbiotroph (0.27%), Pathotroph-Symbiotroph (0.12%), and Saprotroph-Symbiotroph (0.02%). Relative abundance (>1%) of fungal potential functional groups at the level of Guilds were shown in Table S5, but there is no significant difference in all functional groups among the three communities in the two soil layers. PCoA analysis based on the functional assessment data variances among fungal community with the first and second axes explaining 18.43% and 12.54% of the variance, respectively (Figure S1b).

## 4. Discussion

We investigated the bacterial and fungal communities in the mangrove community (MC), the *Spartina alterniflora*–mangrove mixed community (TC), and the *Spartina alterniflora* community (SC) in Zhangjiangkou National Mangrove Wetland Nature Reserve. In this study, we find that the bacterial community diversity indices in the two soil layers show a trend of SC > MC > TC, which is consistent with previous studies on the *Kandelia candel* rhizospheric bacterial community invaded by *Spartina alterniflora* [20]. Soil pH has been proven to be a key factor in regulating soil microbial communities [37,50]. On the one hand, the pH value can select species with compatible growth strategies [28]. On the other hand, studies have shown that acidic soils can inhibit SOM decomposition and C mineralization [29]. Our data show that the TC community has the lowest soil bacterial diversity indices and soil pH and the highest MOC content, which indicate that the invasion of *Spartina alterniflora* may reduce the soil bacterial community diversity by changing the soil pH value, thereby inhibiting C mineralization. However, only the Chao1, Sobs, and Shannon indices in the 15–30 cm soil layer are significantly higher in the SC community than in the TC community, and other differences are not significant (*p* < 0.05). The reason for this phenomenon may be that the invasion of *Spartina alterniflora* affects the soil bacterial diversity of mangroves more obviously in the rhizosphere. For the fungal community, we find that there is no significant difference in the diversity indices of fungi in the two soil layers, but all the diversity indices in the 15–30 cm soil layer show a trend of TC > MC > SC, which is consistent with the significant trend of soil water content in the 15–30 cm soil layer. Research by Yang et al. has shown that the diversity of soil fungal community decreases with the time sequence of *Spartina alterniflora* invasion and has a significant positive correlation with soil moisture and litter biomass. They have also proved that litter biomass is significantly positively correlated with soil moisture [31], which was consistent with our research. PCoA show that the bacterial communities are clearly separated between the two communities, and no significant differences among the three communities are observed in the fungal community, which indicate that the invasion of *Spartina alterniflora* mainly changed the community structure of soil bacteria(Tables S6 and S7).

From different taxonomic levels, the results of LEfSe analysis show significant differences in the relative abundances of bacterial and fungal communities in mangrove community (MC), *Kandelia obovataSpartina alterniflora* transition zone (TC) and the *Spartina alterniflora* community (SC). Our research show that the relative abundance of some bacterial taxa is not significantly different between the MC and SC communities, but different changes have occurred during the invasion process (i.e., TC community). For example, the phylum Acidobacteria and Gemmatimonadetes, the class Alphaproteobacteria decreased sharply, whereas the phylum Proteobacteria and the class Epsilonproteobacteria increased rapidly during the invasion of *Spartina alterniflora*. Previous studies have demonstrated that the relative abundance of Alphaproteobacteria in rhizospheric soil of the mangrove–*Spartina alterniflora* transition zone exhibited sharply decreasing trend after invasion by *Spartina alterniflora*, and that the phylum Acidobacteria has a significant positive correlation with soil pH [20,21], which were consistent with our research. Janssen found that highly nutritious soils lead to greater relative abundance of Alphaproteobacteria [51]. However, Gao indicated that the indicator class of bacteria Alphaproteobacteria was strongly associated with the presence of *Spartina alterniflora* [21], which may explain that the relative abundance of Alphaproteobacteria returned to a higher level after the invasion of *Spartina alterniflora*. Among them, the Epsilonproteobacteria and its members (i.e., the order Campylobacterales, the family Helicobacteraceae, and the genus *Sulfurovum*), which increased significantly during the invasion of *Spartina alterniflora*, belong to the relationship in which the former includes the latter in the order of the level in taxonomy. We infer that the genus *Sulfurovum* plays an important role in the changes of higher classification levels, as it is the dominant group of the class Epsilonproteobacteria. Moreover, some taxa, such as the phylum Nitrospirae, the class Gammaproteobacteria and Nitrospira showed an increasing trend in the later stages of invasion. The order Xanthomonadales, which is the dominant order of the Gammaproteobacteria, contains a single family (Xanthomonadaceae) but many Gram-negative rod-shaped bacteria. The members of this group range from plant and human pathogens to non-pathogenic environmental bacteria, which can survive in harsh conditions such as contaminated soil and hot springs [52].

Different from the results of the bacterial community, the invasion of *Spartina alterniflora* only caused a significant increase in few fungal taxa during the invasion process, and these taxa were at some lower levels (such as family, genus, and species) and classified into the phylum Ascomycota, namely, our results indicate that the response of mangrove soil fungal taxa to *Spartina alterniflora* invasion mainly concentrated on Ascomycota. Ascomycota is a class of fungi that can participate in critical degradation activities [53]. Additionally, a few taxa of Ascomycota are dominant in the global soil fungal community [54]. Moreover, Ascomycetes have been reported as oligotrophic fungi with the ability to withstand stressful environments (e.g., low nutrient availability and drought stress) [55,56,57]. Our previous study also found that the invasion of *Spartina alterniflora* caused an increase in the decomposition of mangrove soil organic carbon, which hindered the accumulation of soil organic carbon [10]. The reduction of organic carbon in mangrove soil after *Spartina alterniflora* invasion may be a large part of the reason for the change of soil microbial community structure. In addition, many taxa with significant changes have been reported as animal, plant, or human pathogens, such as the genus Sporothrix and Phialemonium [58,59]. Therefore, the invasion of *Spartina alterniflora* may alter the physical and chemical properties of soil and the structure of flora and fauna by changing the community structure of soil microorganisms.

Assessment of potential functions using Tax4Fun and FunGuild based on high-throughput sequencing has been applied to analyze possible functions of different microorganisms [31,60]. However, the analysis based on the assessment of bacterial and fungal potential functions does not show significant differences in soil microbial possible functions before and after the invasion of Spartina alterniflora(Tables S8 and S9). We infer that the invasion of *Spartina alterniflora* did not change the ecological possible functions of soil microorganisms in general, but changed taxa with certain ecological functions. The potential functional assessment was based on the classification of OTUs and the SILVA123 database of bacteria and the FUNGuild database of fungi as a reference. In our data, the number of OTUs classified as genera in the bacterial community is very small (<20.16%), and the fungal community has only 833 OTUs that can be classified to the genus level, which are limited by the current development of microbial taxonomy. However, we can still use Tax4Fun and FUNGuild to assess the potential functions of known taxonomic groups of microorganisms under the current development of microbiology, so as to provide reference for the impact of *Spartina alterniflora* invasion on the potential ecological functions of soil microorganisms. In future research, it is necessary to combine more powerful tools (such as metagenomic sequencing and other technologies) or more complete databases to clarify the potential functions of microbial communities.

## 5. Conclusions

This study emphasized the changes of soil microbial communities in the mangrove ecosystem of the Zhangjiang Estuary wetland after the invasion of *Spartina alterniflora*. Our results suggest that although the invasion of *Spartina alterniflora* does not cause significant changes in the diversity of soil bacteria and fungi, it can alter the community structure of soil bacteria. The results of the LEfSe analysis show that the relative abundance of some bacterial taxa is not significantly different between the MC and SC communities, but different changes have occurred during the invasion process (i.e., TC community). Different from the results of the bacterial community, the invasion of *Spartina alterniflora* can only cause a significant increase in few fungal taxa during the invasion process, and these taxa are at some lower levels (such as family, genus and species) and classified into the phylum Ascomycota. Although the invasion of *Spartina alterniflora* changes the taxa with certain ecological functions, it may not change the potential ecological functions of soil microorganisms (i.e., the potential metabolic pathways of bacteria, nutritional patterns, and fungal associations).

## Figures and Tables

**Table 1:** The soil bacterial diversity indices (Chao1, Sobs, Shannon, Simpson) in different communities.

Soil Layer	Plant Community	Chao1	Sobs	Shannon	Simpson (10^−2^)
0–15 cm	SC	2654.3 ± 173.5 a	2224.8 ± 139.1 a	9.5 ± 0.1 a	99.6 ± 0.1 a
	TC	2722.9 ± 22.2 a	2194.6 ± 11.8 a	9.2 ± 0.2 a	99.4 ± 0.2 a
	MC	2673.7 ± 53.3 a	2170.4 ± 76.9 a	9.3 ± 0.2 a	99.5 ± 0.2 a
15–30 cm	SC	2796.5 ± 75.5 a	2322.1 ± 68.4 a	9.5 ± 0.1 a	99.6 ± 0.1 a
	TC	2560.4 ± 51.0 b	2074.6 ± 29.2 b	8.9 ± 0.3 b	99.1 ± 0.6 a
	MC	2633.3 ± 126.6 ab	2123.9 ± 93.9 ab	9.1 ± 0.1 ab	99.3 ± 0.3 a

The values were shown as mean ± standard deviation (*n* = 3). Different letters in the same column indicate significant differences among the plant communities in the same soil layer at a *p* < 0.05 according to Tukey test.

**Table 2:** The soil fungal diversity indices (Chao1, Sobs, Shannon, Simpson) in different communities (there was no significant difference of each diversity index among the three plant communities in the same soil layer at a *p* > 0.05 according to Tukey’s test).

Soil Layer	Plant Community	Chao1	Sobs	Shannon	Simpson (10^−2^)
0–15 cm	SC	251.0 ± 62.1	242.6 ± 54.0	4.7 ± 1.5	79.0 ± 25.2
	TC	306.2 ± 177.4	290.0 ± 178.1	5.8 ± 0.7	95.8 ± 2.0
	MC	461.9 ± 178.1	446.6 ± 168.7	6.2 ± 0.5	95.4 ± 2.1
15–30 cm	SC	257.8 ± 7.9	246.7 ± 14.0	5.1 ± 0.9	85.6 ± 12.7
	TC	325.5 ± 120.3	317.6 ± 114.6	6.5 ± 0.3	96.5 ± 1.9
	MC	282.2 ± 118.7	264.3 ± 122.9	5.2 ± 2.5	89.0 ± 15.3

The values are shown as mean ± standard deviation (*n* = 3).

## Data Availability

Publicly available datasets were analyzed in this study. This data can be found here: http://www.ncbi.nlm.nih.gov/bioproject/689656/PRJNA689656.

## Supplementary Materials

Supplementary materials can be found at https://www.mdpi.com/2076-2607/9/1/138/s1, Table S1: Soil properties from different plant communities, Table S2: The relative abundance of bacterial phyla between SC, TC and MC samples, where 1 represents the 0–15 cm soil layer and 2 represents the 15–30 cm soil layer. Table S3: The relative abundance of fungal phyla between SC, TC and MC communities, where 1 represents the 0–15 cm soil layer and 2 represents the 15–30 cm soil layer. Table S4: Relative abundance (>1%) of bacterial potential functional pathways at level 2 in the SC, TC and MC communities. Table S5: Relative abundance (>1%) of fungal potential functional groups at the level of Guilds in the SC, TC and MC communities (There was no significant difference of each functional group among the three plant communities in the same soil layer at a *p* > 0.05 according to Tukey test). Table S6: Results of PerMANOVA test in bacterial community composition among the SC, TC and MC communities. Table S7: Results of PerMANOVA test in fungal community composition among the SC, TC and MC communities. Table S8: Results of PerMANOVA test in bacterial function composition among the SC, TC and MC communities. Table S9: Results of PerMANOVA test in fungal function composition among the SC, TC and MC communities. Table S10: The barcode information corresponding to samples’ information. Figure S1: Results of PCoA showing the first two principal coordinates that, combined, explain 70.78% and 30.97% of the observed variation in bacterial (a) and fungal (b) functional structure. The shapes of up triangle and square on the figure legend correspond to the soil layer of 0–15 cm and 15–30 cm, and the colors of red, green and yellow correspond to the SC, TC and MC communities, respectively (PerMANOVA test, *p* < 0.05).
