# Supplementary material for: Effects of Spartina alterniflora Invasion on Soil Microbial Community Structure and Ecological Functions
Source: Microorganisms. 2021 Jan 9;9(1):138. doi: 10.3390/microorganisms9010138 (PMC7827921; doi:10.3390/microorganisms9010138)
Supplement: Supplementary file 1 [file microorganisms-09-00138-s001.pdf]

Table S1. Soil properties from different plant communities.

soil layer	Plant community	Sand (%)	Silt+clay (%)	Water content (g/g)	pH	EC (mS/cm)	SOC (g/kg)	POC (g/kg)	MOC (g/kg)
0-15 cm	SC	3.33 ± 0.84b	96.67 ± 1.81a	0.90 ± 0.21a	6.18 ± 0.44a	1.45 ± 0.29b	10.23 ±2.33b	6.97 ± 0.44b	3.26 ± 0.63b
	TC	6.31 ± 2.49b	93.69 ± 3.02a	1.10 ± 0.24a	5.15 ± 0.84b	2.03 ± 0.69a	15.85 ± 2.81a	7.11 ± 0.26b	8.74 ± 1.27a
	MC	15.98 ± 4.34a	84.02 ± 4.41b	0.95 ± 0.15a	5.69 ±0.78ab	2.52 ± 0.63a	16.49 ± 4.96a	14.85 ± 1.6a	1.64 ± 0.26b
15-30 cm	SC	4.08±1.91b	95.92±1.91a	0.83±0.27b	6.09±0.77a	1.84±0.35b	10.21±0.84c	1.31±0.23c	8.90±0.86b
	TC	13.80±3.73a	86.20±21.25a	1.07±0.13a	4.80±0.77b	2.76±0.38a	15.35±0.58b	4.37±0.28b	10.98±0.66b
	MC	11.22±3.42a	88.78±8.14a	0.89±0.12ab	5.56±0.95ab	2.84±0.59a	23.24±6.06a	6.56±1.35a	16.68±6.25a

The values were shown as mean ± standard deviation (n = 3). Different letters in the same column indicate significant differences among the plant communities in the same soil layer at a $p < 0.05$ according to Tukey test. Abbreviation: EC: Electrical conductance, SOC: Soil organic carbon, POC: Particulate organic carbon, MOC: Mineral organic carbon.

Table S2. The relative abundance of bacterial phyla between SC, TC and MC samples, where 1 represents the 0-15cm soil layer and 2 represents the 15–30cm soil layer.

**Phylum**	**OTU**	**Total**	**SC**		**TC**		**MC**	
			**SC1**	**SC2**	**TC1**	**TC2**	**MC1**	**MC2**
Proteobacteria	36.42%	55.31%	55.14%±0.50%	54.69%±1.37%	56.42%±0.55%	56.86%±4.91%	53.90%±2.36%	54.86%±3.45%
Bacteroidetes	9.15%	8.54%	7.40%±1.64%	6.68%±0.72%	9.38%±0.96%	7.51%±1.13%	12.68%±5.64%	7.50%±1.05%
Chloroflexi	7.24%	4.64%	3.07%±0.26%	5.21%±0.82%	3.83%±0.41%	5.90%±1.06%	3.57%±1.17%	6.28%±2.03%
Acidobacteria	5.25%	3.84%	4.63%±1.10%	3.27%±0.40%	3.33%±0.20%	3.32%±0.90%	4.28%±0.14%	4.13%±0.11%
Nitrospirae	1.83%	3.68%	3.93%±0.48%	5.43%±0.67%	3.03%±0.74%	3.60%±0.21%	2.30%±0.39%	3.90%±0.40%
Gemmatimonadetes	2.21%	2.40%	3.47%±0.62%	2.89%±0.17%	1.76%±0.16%	1.77%±0.43%	2.44%±0.41%	2.17%±0.53%
Verrucomicrobia	3.72%	2.39%	2.73%±0.79%	1.69%±0.24%	2.68%±1.00%	1.48%±0.44%	3.51%±0.68%	2.16%±0.18%
Firmicutes	2.79%	2.14%	1.72%±0.92%	1.88%±0.42%	4.33%±2.21%	2.49%±1.72%	1.55%±1.17%	0.80%±0.39%
Planctomycetes	7.06%	1.86%	2.38%±0.34%	2.29%±0.41%	1.35%±0.13%	1.47%±0.10%	1.82%±0.30%	1.85%±0.44%

Ignavibacteriae	1.16%	1.38%	1.33%±0.24%	1.19%±0.16%	1.53%±0.21%	1.33%±0.54%	1.39%±0.36%	1.51%±0.42%
Latescibacteria	2.29%	1.19%	1.39%±0.40%	1.33%±0.25%	0.95%±0.07%	1.11%±0.25%	1.19%±0.22%	1.14%±0.18%
Actinobacteria	1.61%	1.06%	1.07%±0.58%	0.69%±0.12%	1.18%±0.23%	0.67%±0.10%	1.70%±0.63%	0.95%±0.31%
Spirochaetae	2.06%	0.74%	0.58%±0.16%	0.97%±0.23%	0.72%±0.08%	0.92%±0.07%	0.42%±0.12%	0.82%±0.21%
Deferribacteres	0.93%	0.68%	0.49%±0.11%	0.71%±0.12%	0.59%±0.01%	0.90%±0.11%	0.47%±0.17%	0.94%±0.30%
RBG-1 (Zixibacteria)	1.06%	0.51%	0.42%±0.06%	0.64%±0.12%	0.34%±0.04%	0.69%±0.17%	0.37%±0.16%	0.63%±0.12%
Fusobacteria	0.13%	0.49%	0.36%±0.36%	0.17%±0.04%	0.14%±0.04%	0.23%±0.07%	0.62%±0.47%	1.43%±1.79%
Aminicenantes	0.65%	0.46%	0.26%±0.09%	0.66%±0.24%	0.37%±0.09%	0.64%±0.14%	0.27%±0.10%	0.54%±0.06%
Parcubacteria	2.59%	0.33%	0.35%±0.06%	0.33%±0.03%	0.34%±0.10%	0.39%±0.05%	0.26%±0.05%	0.28%±0.05%
Cyanobacteria	0.25%	0.30%	0.73%±0.66%	0.21%±0.11%	0.30%±0.06%	0.10%±0.03%	0.33%±0.11%	0.17%±0.07%
Nitrospinae	0.33%	0.29%	0.54%±0.23%	0.37%±0.12%	0.19%±0.06%	0.17%±0.08%	0.34%±0.10%	0.17%±0.06%
Chlorobi	0.25%	0.18%	0.23%±0.12%	0.15%±0.06%	0.18%±0.03%	0.13%±0.03%	0.18%±0.09%	0.18%±0.06%
AC1	0.20%	0.17%	0.15%±0.07%	0.30%±0.05%	0.10%±0.01%	0.19%±0.09%	0.12%±0.08%	0.15%±0.04%
Lentisphaerae	0.48%	0.16%	0.29%±0.14%	0.22%±0.13%	0.14%±0.01%	0.11%±0.02%	0.11%±0.04%	0.09%±0.03%
Peregrinibacteria	0.35%	0.11%	0.14%±0.02%	0.19%±0.04%	0.10%±0.05%	0.10%±0.04%	0.07%±0.01%	0.09%±0.03%
Unclassified and Others	10.00%	7.14%	7.18%±0.49%	7.84%±0.81%	6.72%±1.19%	7.90%±0.90%	6.10%±1.03%	7.24%±0.13%

The values were shown as mean ± standard deviation (*n* = 3, except for OTU and Total *n* = 18).

Table S3. The relative abundance of fungal phyla between SC, TC and MC communities, where 1 represents the 0–15cm soil layer and 2 represents the 15–30cm soil layer.

Phylum	OTU	Total	SC		TC		MC	
			SC1	SC2	TC1	TC2	MC1	MC2
Ascomycota	24.44%	40.36%	38.51%±24.95%	47.67%±33.79%	38.83%±19.91%	39.96%±26.22%	33.65%±23.45%	41.53%±20.98%
Basidiomycota	7.32%	5.61%	0.92%±0.47%	4.14%±2.68%	10.51%±9.30%	6.72%±3.96%	6.23%±5.41%	5.02%±2.34%
Chytridiomycota	0.36%	0.39%	1.73%±2.39%	0.08%±0.08%	0.32%±0.23%	0.05%±0.05%	0.12%±0.10%	0.02%±0.03%
Zygomycota	0.20%	0.02%	0.05%±0.01%	0.00%±0.00%	0.00%±0.00%	0.00%±0.00%	0.00%±0.00%	0.05%±0.07%
Unclassified	67.68%	53.62%	58.79%±26.32%	48.10%±34.86%	50.34%±24.07%	53.26%±30.20%	60.00%±28.42%	53.38%±21.16%

The values were shown as mean ± standard deviation (*n* = 3, except for OTU and Total *n* = 18).

Table S4. Relative abundance (>1%) of bacterial potential functional pathways at level 2 in the SC, TC and MC communities.

soil layer	Pathway (level 1)	Pathway (level 2)	SC（%）	TC（%）	MC（%）
0–15cm	Cellular Processes	Cell motility	3.16±0.10	3.01±0.11	2.47±0.71
		Cell growth and death	1.96±0.06	1.90±0.06	1.85±0.13
		Cellular community. prokaryotes	0.45±0.02	0.45±0.02	0.43±0.04
		Transport and catabolism	0.30±0.02	0.29±0.02	0.41±0.15
	Environmental Information Processing	Membrane transport	9.22±0.20	9.40±0.16	8.78±0.68
		Signal transduction	9.22±0.53	9.41±0.29	8.63±0.52
	Genetic Information Processing	Translation	5.21±0.06	5.08±0.01	5.21±0.10
		Replication and repair	4.72±0.07a	4.52±0.03b	4.72±0.11a
		Folding. sorting and degradation	2.61±0.03	2.60±0.01	2.74±0.12
	Human Diseases	Infectious diseases Bacterial	1.18±0.00	1.21±0.01	1.20±0.04
	Metabolism	Carbohydrate metabolism	12.29±0.14	12.90±0.75	13.51±1.17
		Amino acid metabolism	11.19±0.30	11.12±0.14	11.21±0.10
		Energy metabolism	8.69±0.31	8.60±0.16	8.10±0.58
		Metabolism of cofactors and vitamins	7.78±0.08a	7.53±0.07b	7.71±0.06a
		Nucleotide metabolism	5.65±0.07	5.59±0.03	5.69±0.10
		Glycan biosynthesis and metabolism	3.12±0.07	3.04±0.14	3.56±0.54
		Xenobiotics biodegradation and metabolism	3.19±0.04	3.20±0.12	3.13±0.12
		Lipid metabolism	2.89±0.06	2.91±0.01	3.11±0.30
		Metabolism of terpenoids and polyketides	2.51±0.12	2.52±0.11	2.44±0.02
		Metabolism of other amino acids	2.01±0.05	2.06±0.02	2.17±0.13
		Biosynthesis of other secondary metabolites	0.80±0.03	0.79±0.03	0.85±0.09

15–30cm	Organismal Systems	Endocrine system	0.32±0.02b	0.37±0.00ab	0.39±0.05a
	Cellular Processes	Cell motility	3.23±0.13	3.30±0.22	3.14±0.37
		Cell growth and death	1.98±0.01	2.00±0.04	1.98±0.07
		Cellular community. prokaryotes	0.43±0.00	0.42±0.07	0.42±0.03
		Transport and catabolism	0.28±0.02	0.30±0.03	0.30±0.07
	Environmental Information Processing	Signal transduction	9.63±0.32	9.72±1.01	9.21±0.40
		Membrane transport	9.12±0.23	9.06±0.74	8.85±0.20
	Genetic Information Processing	Translation	5.29±0.14	5.27±0.47	5.37±0.25
		Replication and repair	4.77±0.13	4.61±0.26	4.67±0.11
		Folding. sorting and degradation	2.66±0.06	2.74±0.10	2.74±0.02
		Transcription	0.23±0.01	0.23±0.01	0.23±0.01
	Human Diseases	Infectious diseases Bacterial	1.17±0.04	1.17±0.10	1.13±0.07
		Neurodegenerative diseases	0.14±0.00	0.14±0.00	0.14±0.01
	Metabolism	Carbohydrate metabolism	12.49±0.39	12.84±0.70	13.00±0.20
		Amino acid metabolism	10.98±0.30	10.76±0.16	10.85±0.16
		Energy metabolism	8.64±0.16	8.78±0.35	8.83±0.48
		Metabolism of cofactors and vitamins	7.69±0.10	7.57±0.42	7.79±0.19
		Nucleotide metabolism	5.69±0.12	5.58±0.42	5.72±0.19
		Glycan biosynthesis and metabolism	3.16±0.11	3.10±0.35	3.20±0.10
		Xenobiotics biodegradation and metabolism	3.02±0.08	3.05±0.23	3.09±0.14
		Lipid metabolism	2.81±0.03	2.83±0.28	2.77±0.27
		Metabolism of terpenoids and polyketides	2.32±0.03	2.29±0.18	2.34±0.10
		Metabolism of other amino acids	1.99±0.01	1.97±0.13	1.94±0.16

	Biosynthesis of other secondary metabolites	0.79±0.04	0.74±0.03	0.74±0.05
Organismal Systems	Endocrine system	0.34±0.04	0.40±0.04	0.37±0.03
	Environmental adaptation	0.26±0.00	0.26±0.01	0.27±0.01
	Digestive system	0.23±0.03	0.20±0.07	0.23±0.01
	Nervous system	0.13±0.00	0.13±0.01	0.13±0.01

The values were shown as mean ± standard deviation (n = 3). Different letters in the same row indicate significant differences among the plant communities in the same soil layer at a $p < 0.05$ according to Tukey test.

Table S5. Relative abundance (>1%) of fungal potential functional groups at the level of Guilds in the SC, TC and MC communities (There was no significant difference of each functional group among the three plant communities in the same soil layer at a p>0.05 according to Tukey test).

soil layer	Trophic Mode	Fun Guild	MC（%）	TC（%）	SC（%）
0–15cm	Pathotroph	Plant Pathogen	3.08±2.58	2.90±3.80	1.44±0.83
	Pathotroph-Saprotroph	Plant Pathogen_Undefined Saprotroph	0.69±0.54	1.47±1.71	0.84±0.62
		Endophyte_Lichen Parasite_Plant Pathogen_Undefined Saprotroph	0.14±0.17	0.17±0.29	1.11±1.87
	Pathotroph-Saprotroph-Symbiotroph	Animal Pathogen_Endophyte_Lichen Parasite_Plant Pathogen_Soil Saprotroph_Wood Saprotroph	0.66±0.90	6.42±7.03	0.95±1.18
		Fungal Parasite_Undefined Saprotroph	0.74±0.11	3.28±2.85	0.03±0.06
		Endomycorrhizal_Plant Pathogen_Undefined Saprotroph	0.00±0.00	2.29±3.96	0.00±0.00
		Animal Pathogen_Endophyte_Epiphyte_Plant Pathogen_Undefined Saprotroph	0.00±0.00	1.02±1.77	0.00±0.00
	Saprotroph	Undefined Saprotroph	9.37±6.58	8.68±6.09	5.55±4.11
		Leaf Saprotroph	3.18±5.02	0.94±1.58	0.00±0.00
15–30cm	Pathotroph	Plant Pathogen	2.57±3.91	3.28±3.20	2.82±1.49
	Pathotroph-Saprotroph	Plant Pathogen_Undefined Saprotroph	1.37±1.90	2.48±1.89	11.09±18.39
		Endophyte_Lichen Parasite_Plant Pathogen_Undefined Saprotroph	0.03±0.05	0.47±0.46	3.63±5.56

	Animal Endosymbiont_Animal Pathogen_Endophyte_Plant Pathogen_Undefined Saprotroph	0.06±0.10	1.08±1.29	0.29±0.29
Pathotroph-Saprotroph-Symbiotroph	Animal Pathogen_Endophyte_Lichen Parasite_Plant Pathogen_Soil Saprotroph_Wood Saprotroph	1.91±3.29	1.41±1.83	1.02±1.32
	Animal Pathogen_Endophyte_Plant Pathogen_Wood Saprotroph	2.55±4.42	0.13±0.22	0.62±1.07
	Fungal Parasite_Undefined Saprotroph	0.46±0.74	0.54±0.88	1.61±2.20
	Animal Pathogen_Endophyte_Epiphyte_Undefined Saprotroph	0.10±0.16	0.03±0.06	1.02±1.46
Saprotroph	Undefined Saprotroph	7.02±7.74	8.66±6.39	12.62±14.83
	Leaf Saprotroph	0.81±0.87	3.03±3.26	0.04±0.06
Symbiotroph	Ectomycorrhizal	0.63±1.08	0.18±0.30	0.39±0.67

The values were shown as mean ± standard deviation (n = 3).

Table S6. Results of PerMANOVA test in bacterial community composition among the SC, TC and MC communities.

Group	Distance	Df	Sums of squares	Mean squares	F.Model	Variation (R2)	Pr (>F)	P_adj_BH
MC/TC	Bray-Curtis	1	0.11374649	0.11374649	1.217618645	0.108545199	0.255	0.255
MC/SC	Bray-Curtis	1	0.255615679	0.255615679	2.875526269	0.223332717	0.004	0.006
TC/SC	Bray-Curtis	1	0.285317863	0.285317863	3.340142551	0.250382823	0.002	0.006

Table S7. Results of PerMANOVA test in fungal community composition among the SC, TC and MC communities.

group	distance	Df	Sums of squares	Mean squares	F.Model	Variation (R2)	Pr (>F)	P_adj_BH
MC/TC	Bray-Curtis	1	0.306614438	0.306614438	0.753555514	0.07007501	0.769	0.769
MC/SC	Bray-Curtis	1	0.597120908	0.597120908	1.488905006	0.129595031	0.115	0.345
TC/SC	Bray-Curtis	1	0.449499901	0.449499901	1.137566782	0.102137819	0.301	0.4515

Table S8. Results of PerMANOVA test in bacterial function composition among the SC, TC and MC communities.

group	distance	Df	Sums of squares	Mean squares	F.Model	Variation (R2)	Pr (>F)	P_adj_BH
MC/TC	Bray-Curtis	1	0.001219293	0.001219293	1.129237227	0.101465824	0.315	0.391
MC/SC	Bray-Curtis	1	0.001572312	0.001572312	2.141154265	0.176355083	0.063	0.189
TC/SC	Bray-Curtis	1	0.000679518	0.000679518	1.04675786	0.09475702	0.391	0.391

Table S9. Results of PerMANOVA test in fungal function composition among the SC, TC and MC communities.

group	distance	Df	Sums of squares	Mean squares	F.Model	Variation (R2)	Pr (>F)	P_adj_BH
MC/TC	Bray-Curtis	1	0.395107256	0.395107256	1.03024948	0.093402192	0.349	0.5235
MC/SC	Bray-Curtis	1	0.622197008	0.622197008	1.638185441	0.140759524	0.028	0.084
TC/SC	Bray-Curtis	1	0.356267082	0.356267082	0.93447782	0.085461586	0.551	0.551

Table S10. The barcode information corresponding to samples' information.

16S		ITS	
Sample	Barcode	Sample	Barcode
SC1-1	ATCACG	SC1-1	ATCACG
SC2-1	CGATGT	SC2-1	CGATGT
SC3-1	TTAGGC	SC3-1	TTAGGC
SC1-2	TGACCA	SC1-2	TGACCA
SC2-2	ACAGTG	SC2-2	ACAGTG
SC3-2	GCCAAT	SC3-2	GCCAAT
TC1-1	GTTTCG	TC1-1	GTTTCG
TC2-1	ACTTGA	TC2-1	ACTTGA
TC3-1	GATCAG	TC3-1	GATCAG
TC1-2	TAGCTT	TC1-2	TAGCTT
TC2-2	GGCTAC	TC2-2	GGCTAC
TC3-2	CTTGTA	TC3-2	CTTGTA

MC1-1	AGTCAA	MC1-1	AGTCAA
MC2-1	AGTTCC	MC2-1	AGTTCC
MC3-1	ATGTCA	MC3-1	ATGTCA
MC1-2	CGTACG	MC1-2	CGTACG
MC2-2	GTAGAG	MC2-2	GTAGAG
MC3-2	GTCCGC	MC3-2	GTCCGC

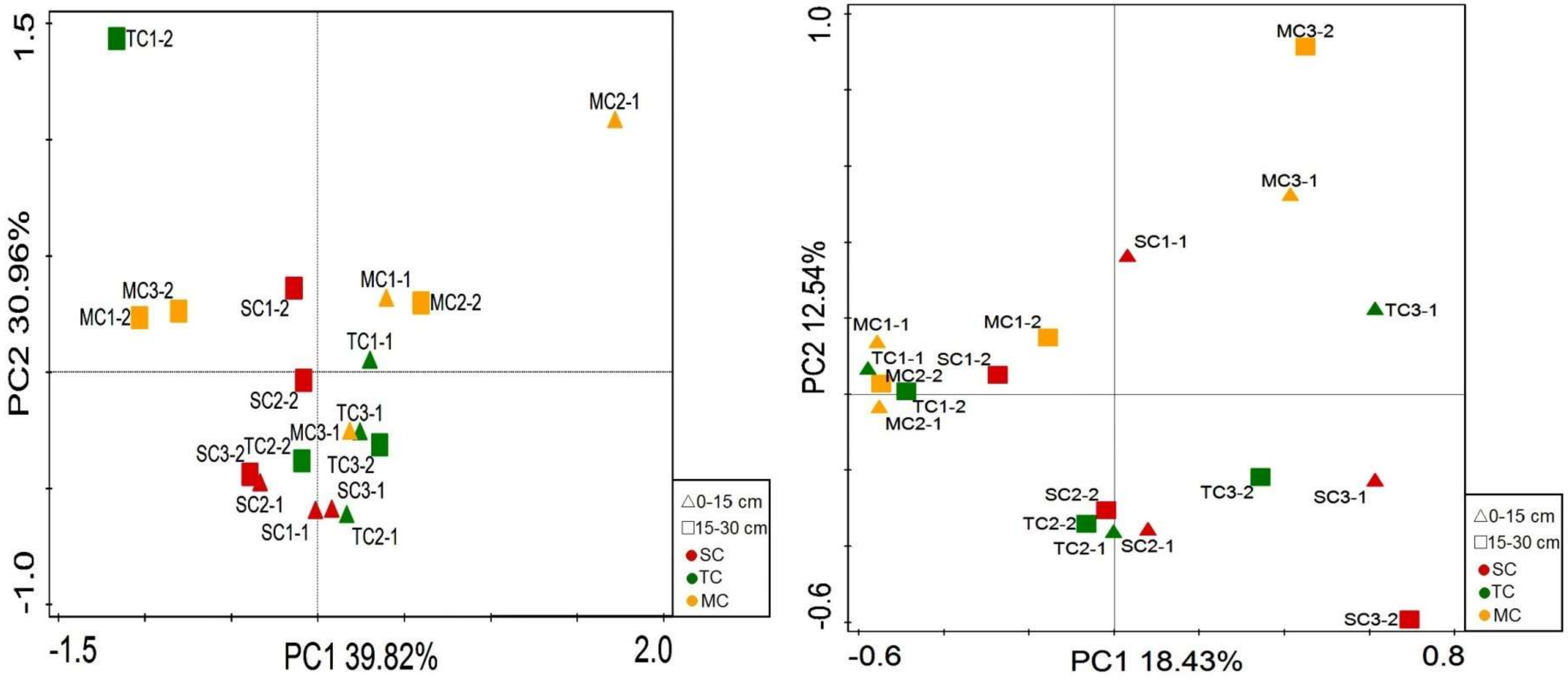

(a) (b)

Figure S1. Results of PCoA showing the first two principal coordinates that, combined, explain 70.78% and 30.97% of the observed variation in bacterial (**a**) and fungal (**b**) functional structure. The shapes of up triangle and square on the figure legend correspond to the soil layer of 0-15 cm and 15–30 cm, and the colors of red, green and yellow correspond to the SC, TC and MC communities, respectively (PerMANOVA test, $p < 0.05$).
